# The perfect storm: respiratory viral surges and anti-infective shortages

**DOI:** 10.1017/ash.2023.160

**Published:** 2023-05-04

**Authors:** Alan E. Gross, Sarah Kabbani, Jennifer Blumenthal

**Affiliations:** 1 Department of Pharmacy Practice, University of Illinois at Chicago College of Pharmacy, Chicago, Illinois; 2 Hospital Pharmacy Services, University of Illinois Hospital and Health Sciences System, Chicago, Illinois; 3 Division of Healthcare Quality Promotion, Centers for Disease Control and Prevention, Atlanta, Georgia; 4 Division of Critical Care Medicine, Department of Anesthesiology, Critical Care and Pain Medicine, Boston Children’s Hospital, Boston, Massachusetts; 5 Division of Infectious Diseases, Department of Pediatrics, Boston Children’s Hospital, Boston, Massachusetts; 6 Department of Anaesthesia, Harvard Medical School, Boston, Massachusetts

In late 2022, ∼40% of US households had a family member with influenza, respiratory syncytial virus (RSV), and/or severe acute respiratory coronavirus virus 2 (SARS-CoV-2).^1^ Increased incidence of influenza and RSV infection occurred much earlier than in the typical respiratory virus season, with the peak occurring in November.^2–4^ Furthermore, RSV and influenza-related hospitalization rates were higher than in the past 5 years. This RSV peak coincided with a SARS-CoV-2 peak in early December 2022.^2^ The overlap in respiratory viral disease burden in the United States contributed to significant healthcare resource utilization in outpatient and inpatient settings for patients with respiratory infection syndromes, especially in pediatric populations, contributing to shortages in anti-infectives and antipyretics.

## Causes of anti-infective shortages

Anti-infective drug shortages continue to occur and are due to decreases in supply and/or increases in demand. Decreases in supply may occur due to raw material shortages or manufacturing issues that may be related to failure to meet current good manufacturing practices or competition among products for the same manufacturing line; these problems are compounded when there is only a single or limited number of manufacturers of the product.^5^ Increases in demand occur because of shortages of an alternative product, due to changes in standards of care, and/or from unexpected increases in the incidence of a disease. Since late 2022, increased demand has been an important contributing factor to the reported shortages of amoxicillin suspension, oseltamivir, and pediatric analgesics given the unusually high incidence of respiratory viral infections beyond what is seen in a typical year.^6^

## Patient harm related to anti-infective shortages

Drug shortages can limit appropriate treatment options and have a potentially adverse impact on patient outcomes. When first-line treatment options are unavailable, clinicians are forced to prescribe alternatives that may be less effective, have broader antimicrobial coverage, or are associated with higher rates of adverse drug events. In the recent amoxicillin shortage, amoxicillin-clavulanate was used for the treatment of acute otitis media or community-acquired pneumonia. In addition to broader coverage, amoxicillin-clavulanate is associated with more gastrointestinal side effects including *Clostridioides difficile* infections (CDIs) compared to amoxicillin.^7,8^ Previous anti-infective shortages led to increased prescribing of agents with higher CDI risk and a subsequent increased rate of CDI reported.^9^ Oseltamivir lessens time to symptom improvement for influenza and prevents influenza infection in those who have been exposed via household contacts.^10^ Oseltamivir shortages reported with increasing influenza rates further limit the ability to prevent and treat the infection, potentially leading to increased disease burden.

## Local and national drug-shortage resources

Multiple national resources help communicate information related to drug shortages. The Food and Drug Administration (FDA) monitors drug shortages, liaises with manufacturers to anticipate and mitigate drug shortages, and provides specific recommendations regarding mitigation strategies.^6^ For example, in January 2023 the FDA provided guidance on compounding certain ibuprofen oral suspension products given the ongoing shortage of commercially available products.^11^ The FDA may also allow the importation of international products during protracted drug shortages. In addition, the American Society of Health-System Pharmacists maintains an updated database and public-facing website of drug shortage information developed and maintained by the University of Utah Drug Information Service.^12^ The site lists current shortages with additional, up-to-date information including known causes, potential resolution dates, proposed mitigation strategies, and links to other related resources. Healthcare providers and the public may also receive explicit guidance from state and local health departments or the Centers for Disease Control and Prevention (CDC) to address drug shortages. For example, the CDC provided comprehensive guidance for inpatient and outpatient facilities to prioritize the use of oseltamivir while supply was limited.^13^ At the state level, the Massachusetts Department of Health along with the Massachusetts chapter of the American Academy of Pediatrics relayed official recommendations to remind caregivers that low-grade fevers are generally not dangerous and to provide guidance on alternative methods for fever management at home.^14^

## Mitigation of anti-infective shortages

Optimizing antimicrobial use is a critical component of mitigation strategies during drug shortages. Antibiotics are commonly prescribed when not needed for viral respiratory infections, and an estimated 28% of all antibiotic prescriptions are unnecessary.^15^ Antimicrobial stewardship strategies, such as active monitoring or delayed prescribing for specific conditions, can be re-emphasized to improve antibiotic prescribing during drug shortages.^16^ When antibiotics are indicated, they are often prescribed for longer durations than recommended.^17^ Prescribing antibiotics for the shortest effective duration can help mitigate drug shortages by conserving available supply. Importantly, promoting the principles of antimicrobial stewardship may help prevent drug shortages from occurring at all. Providing patient educational materials in outpatient settings related to the appropriate use of antibiotics can reduce inappropriate prescribing in general and during peaks in respiratory virus activity.^18^ Routine vaccinations decrease the burden of disease and associated antimicrobial prescribing. All clinicians should encourage annual influenza vaccinations among patients >6 months and uptake of recommended coronavirus disease 2019 (COVID-19) vaccination. In most areas across the United States, significant opportunities exist for increasing vaccination rates.

**Figure 1.**
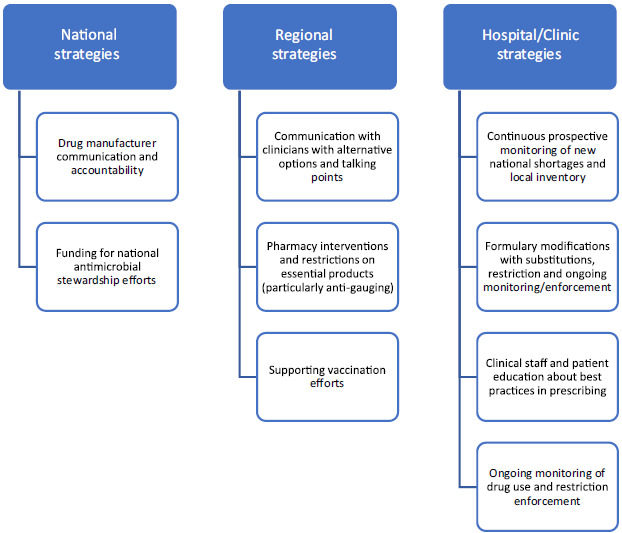
Proposed national and local strategies for improving drug availability during respiratory viral surges.

When shortages of anti-infectives occur, healthcare institutions should use a systematic approach that engages local experts and stakeholders to develop and operationalize a thoughtful response.^19^ Antimicrobial stewardship programs are integral to develop and enact mitigation strategies. Successful collaborations include infectious diseases physicians, infectious diseases pharmacists, administrative leaders. and potentially microbiology and information technology. Response to shortages often include formulary modifications such as medication restrictions or substitutions of alternative agents, which typically require review and approval from institutional pharmacy and therapeutics committees. A mechanism for rapid review and approval of mitigation strategies is essential during anti-infective shortages. Multimodal engagement and education of both frontline providers and patients should succinctly provide recommended substitutions and/or patient prioritization with a clear rationale to improve prescribing practices. Active monitoring of available stock and use of agents in shortage helps predict when additional mitigation strategies must be employed. Importantly, institutions should communicate with leaders in state health departments, suppliers, and local peer institutions because the dynamic factors during shortages may be more local than national. For example, the FDA did not officially classify oseltamivir as being in short supply in 2022, but institutions reported limited supply due to local disease burden and specific contracting wholesalers.^6^ This situation highlights an opportunity for healthcare system pharmacy departments to maintain close communication with their wholesaler(s) and establish contingency supply chains to ensure product availability and potentially collaborate with local peer institutions to share limited supply if feasible. Figure 1 describes potential approaches to mitigating shortages during respiratory viral surges.

In conclusion, as healthcare systems and institutions begin to prepare for respiratory viral season and coincidentally plan activities related to US Antibiotic Awareness Week each year, it may also be a good time to assess product availability and review mitigation strategies to reduce the impact of potential drug shortages.^20^ Given the possibility of continued overlapping burden of these respiratory viruses in future years, having systems in place to anticipate and address shortages is a vital component of patient safety.
